# Intradural Extramedullary Spinal Cord Malignancy Treated With Percutaneous Image-Guided Cryoablation

**DOI:** 10.7759/cureus.100300

**Published:** 2025-12-28

**Authors:** Patrick E Sewell, Miguel Carillo, Manuel Ramiriz, Guillermno Vergel

**Affiliations:** 1 Interventional Oncology, Minimally Invasive Surgery, Radiologica Centro de Diagnóstico por Imagen, Monterrey, MEX; 2 Interventional Radiology, Radiologica Centro de Diagnóstico por Imagen, Monterrey, MEX; 3 Oncology, Radiologica Centro de Diagnóstico por Imagen, Monterrey, MEX; 4 Intensive Care Unit, Radiologica Centro de Diagnóstico por Imagen, Monterrey, MEX

**Keywords:** cryoablation, extramedullary, intradural, sarcoma, teratoma

## Abstract

A 63-year-old male with several months of bilateral gluteal radicular pain and changes in bowel and bladder function presented with an acute onset of cauda equina syndrome. Evaluation and diagnostic imaging revealed an intradural extramedullary spinal tumor at L4. The patient’s symptoms were severe enough to require the use of a wheelchair and high doses of narcotic pain medication. Over the next 18 months, he would suffer from persistent bilateral gluteal and lower extremity radiculopathy, paresthesia, lower extremity weakness and paralysis, erectile dysfunction, bowel sphincter and bladder sphincter dysfunction, and disequilibrium. Initially, the tumor was resected, and the initial cytopathology impression was felt to be in error, resulting in a second opinion analysis, as well as a second tumor biopsy, generating a third cytopathological report. The three cyto-pathology reports were conflicting, with the analysis ranging from benign to malignant tumors. The final cytopathological diagnosis was indicative of a malignant teratoma with sarcoma transformation. The second recurrence was treated with chemotherapy, followed by spinal artery immunotherapy embolization. Both treatments failed. The rapid recurrence was suggestive of somatic malignant transformation within the tumor. CT-guided percutaneous cryoablation achieved a cure with no residual viable tumor, and the patient remains cancer-free one year post cryoablation. Over the months following cryoablation, the patient's neurological system recovered. At one year post-cryoablation, the patient is now pain-free without medication, fully ambulatory, and has had some return of bowel function. He has returned to work as a physician and clinic administrator.

## Introduction

Spinal cord tumors comprise 2-4% of central nervous system malignancies, with intradural extramedullary types, about 40%, including meningiomas, schwannomas, and rare teratomas that may cause cauda equina syndrome when in the lumbar region [1-5].

Intradural extramedullary teratomas are exceptionally rare entities, with only 146 cases reported in the medical literature to date [6]. Within this limited cohort, only 12 cases have been documented in patients over 60 years of age [7]. The rarity increases exponentially when considering malignant transformation within these tumors, as benign teratomas have only a 2% risk of malignant transformation [8].

The management of intradural extramedullary tumors has traditionally relied on surgical resection as the primary treatment modality [9]. However, the intimate relationship between these tumors and neural structures often precludes complete resection, leading to recurrence rates of 9-11% even after gross total resection [10].

For spinal teratomas exhibiting malignant histological features or demonstrating recurrence after surgery, adjuvant therapies such as radiation and chemotherapy have been employed, though with variable and often limited success [6,11,12]. Allsopp et al. reported that postoperative radiotherapy should be considered when malignant germ-cell components or immature elements are present, even when gross total excision appears complete [13-15]. Similarly, Li et al. noted that radiotherapy is justified in cases with malignant differentiation, although chemotherapy outcomes remain inconsistent [9]. Han et al. described a recurrent cervical immature teratoma managed with combined surgery, radiotherapy, and chemotherapy, highlighting the difficulty of achieving durable remission [16]. Guo et al. further emphasized that, when malignant transformation is identified in adult teratomas, treatment paradigms must shift toward multimodal therapy incorporating adjuvant chemoradiation. Collectively, these reports underscore the uncertain benefit yet clinical rationale for adjuvant treatment in aggressive or recurrent spinal teratomas [17].

In recent years, minimally invasive ablative techniques have emerged as alternative treatment options for various malignancies [11]. Among these, cryoablation has shown particular promise in the treatment of neural tumors due to its unique property of preserving myelin sheaths while destroying axons, potentially allowing for neural regeneration [12]. This characteristic distinguishes cryoablation from other thermal ablative techniques and may offer superior functional outcomes in the treatment of spinal tumors [13].

To our knowledge, this represents the first reported case of an intradural extramedullary spinal teratoma with malignant transformation and, more remarkably, the first successful treatment of such a lesion using percutaneous cryoablation. This case highlights both the diagnostic challenges associated with these rare tumors and the potential role of innovative treatment approaches in managing complex spinal neoplasms.

## Case presentation

A 63 y/o male physician gave a five- to six-year history of altered bowel and bladder pattern with urgency and a one-month history of radicular buttock pain. In late October 2021, he developed cauda equina syndrome with progressive bilateral radiculopathy, loss of balance, urinary retention, erectile dysfunction, and bowel incontinence. MRI L-spine with contrast dated November 24, 2021, demonstrated a cauda equina mass at the L4 level measuring 3.2 cm × 1.7 cm × 1.4 cm (3.99 cm³).

On December 9, 2021, a complete and total surgical resection of the lumbar tumor was performed at Stanford University Medical Center in Palo Alto, CA. Initial pathological analysis of the resected mass was felt to be consistent with a mature teratoma. After his initial resection, his symptoms were significantly improved but began to rapidly return.

Post-operative MRI L-spine with contrast was obtained on January 10, 2022, one month after his initial resection. It demonstrated recurrence of the mass. The rapid recurrence suggested somatic malignancy arising out of the teratoma; thus, a second opinion analysis of the first resected tumor specimen was requested. The recurrent cauda equina tumor measured 1.7 cm × 1.7 cm × 2.4 cm (3.63 cm³) on the MRI L-spine dated January 10, 2022.

On January 14, 2022, the patient presented emergently to Cedars-Sinai Medical Center in Los Angeles, CA, again suffering from bowel and bladder incontinence with lower extremity pain and numbness. These symptoms were consistent with recurrent spinal tumor and worsening cauda equina syndrome. Emergently obtained MRI L-spine from January 14, 2022, demonstrated a large hemorrhagic tumor at L4/5 measuring 4.89 cm in greatest dimension. The MRI exam images showed a tumor that appeared much worse than the preoperative MRI of November 24 and the MRI from four days prior, dated January 10, 2022. The increase in size from 3.63 cm in the greatest diameter was secondary to acute hemorrhage within the mass.

A second lumbar tumor total resection was performed emergently at Cedars-Sinai Medical Center in Los Angeles, CA, on January 14, 2022. Cytopathological analysis of the 4.89 cm second tumor resection was interpreted as a malignant non-seminomatous germ cell tumor with a predominance of immature teratoma.

On January 25, 2022, the second opinion pathology report of the initially surgically resected spinal tumor was reclassified as a nonmature germ cell tumor with predominant immature teratoma features and transformation to sarcoma not otherwise specified (NOS). Microsatellite instability was noted to be negative.

The tumor was treated as a germ cell tumor with sarcomatous transformation of a teratoma. For the week of February 11-February 15, 2022, the patient received his first cycle of EP (etoposide and cisplatin) chemotherapy.

On February 24, 2022, an MRI of the L-spine with contrast demonstrated the cauda equina tumor to have increased in size and measured 4.4 cm × 1.9 cm × 1.8 cm (7.88 cm³). The following day, on February 25, 2022, the chemotherapy regimen was changed to doxorubicin and ifosfamide with mesna.

On March 4, 2022, the chemotherapy was changed back to EP. Chemotherapy was subsequently discontinued due to a lack of response. No other chemotherapy was offered.

Regarding chemotherapy versus radiation therapy, it was felt that he would not be able to tolerate chemotherapy after radiation therapy due to expected bone marrow suppression from the radiation therapy. It was also felt that chemotherapy had a better chance of achieving a cure. Radiation therapy could delay tumor growth for a brief period, but at a significant risk of bone marrow suppression, preventing the chance of receiving chemotherapy afterward. After chemotherapy, the risk-benefit of radiation therapy was felt to be unacceptable.

After chemotherapy, the patient was followed clinically for the next eight months as his neurological deficits worsened and his pain increased, requiring higher and higher doses of narcotic pain medication.

**Figure 1:**
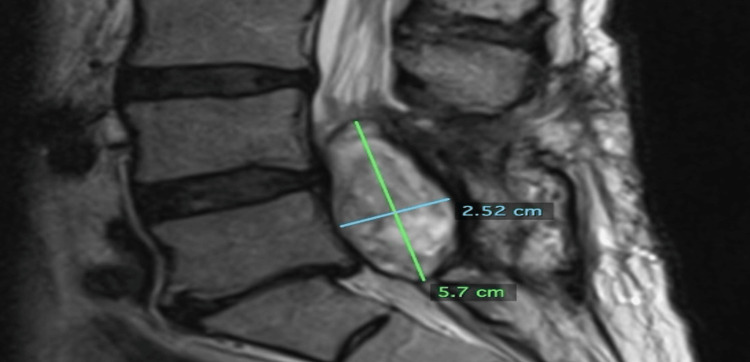
Sagittal image from an MRI L-spine with contrast dated November 16, 2022. The tumor measured 5.7 cm superiorly to inferiorly and 2.52 cm anteriorly to posteriorly.

**Figure 2:**
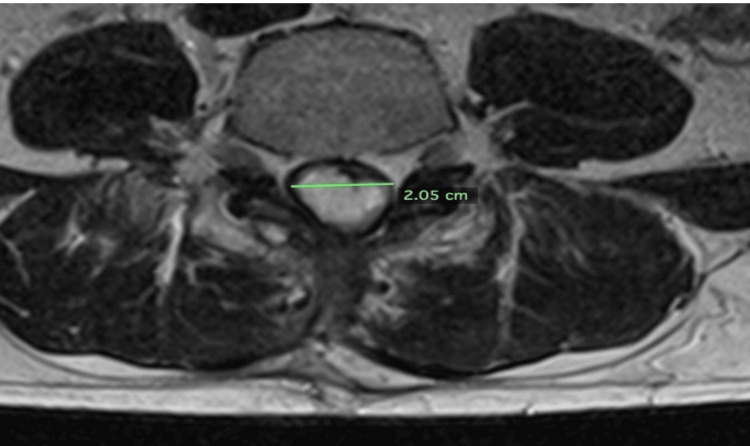
Axial image from an MRI L-spine with contrast dated November 16, 2022. The tumor measured 2.05 cm transversely.

On November 16, 2022, an MRI L-spine with contrast demonstrated the tumor to now measure 5.7 cm x 2.52 cm x 2.05 cm (15.42 cm^3^) (Figures 1-2).

In late November 2022, interventional radiology was consulted and offered an outpatient lumbar spinal artery arteriogram with intra-arterial immunotherapy, and bland particle embolization was offered. On December 17, 2022, the patient underwent a right L4 artery and a right L5 lumbar artery immuno-embolization, as well as the right L5 anterior and posterior radicular artery immuno-embolization in Scottsdale, AZ. A total dose of 120 mg of nivolumab was mixed with 250-micron Embozene particles and delivered to the tumor capillary bed without complications.

Five weeks later, a post-embolization MRI L-spine with contrast, dated January 23, 2023, was obtained and demonstrated a decrease in tumor dimensions; however, viable tumor was evident. The mass measured 2.1 cm x 4.1 cm x 2.3 cm (10.3 cm^3^).

**Figure 3:**
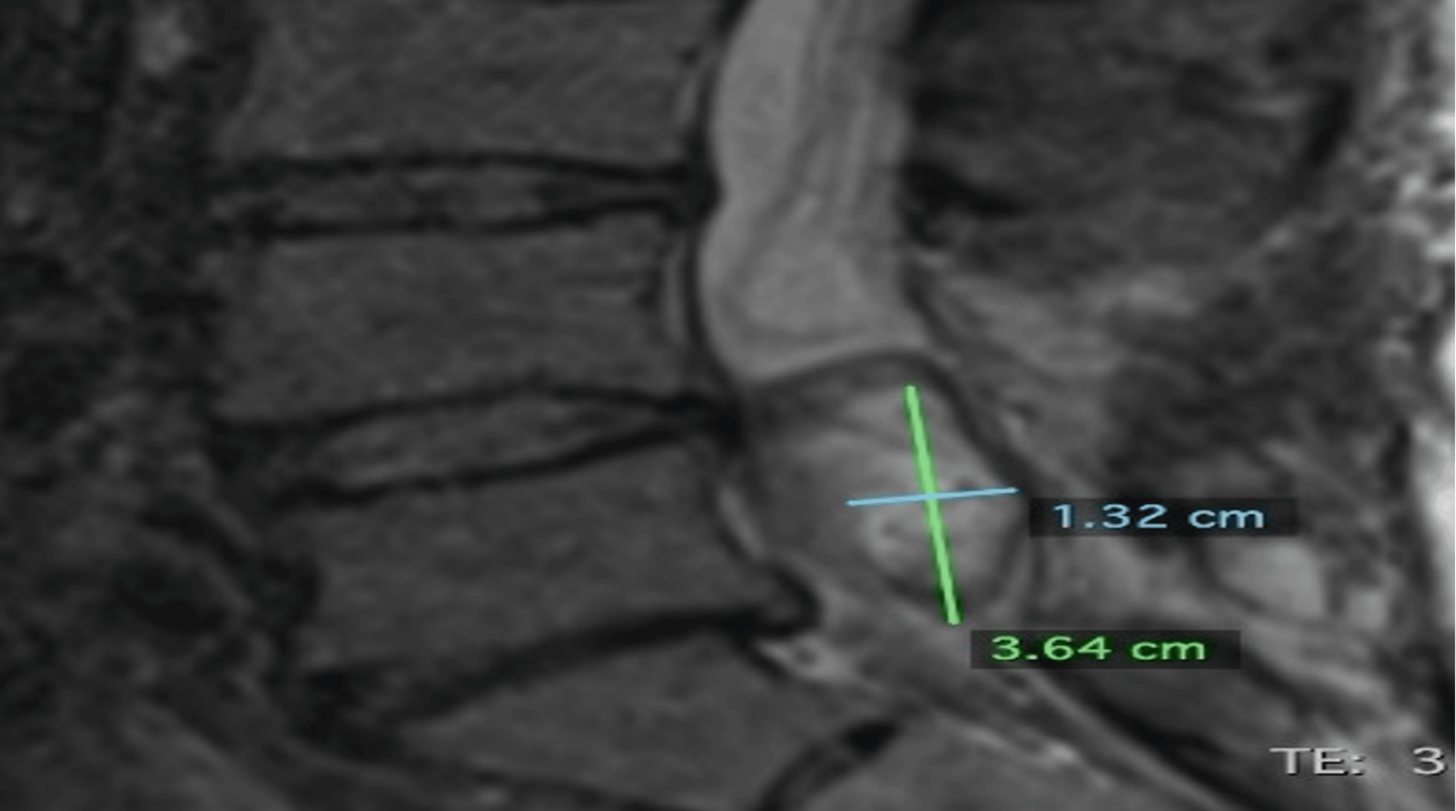
Pre-cryoablation sagittal image from an MRI dated May 3, 2023. The tumor measured 3.64 cm superiorly to inferiorly and 1.32 cm anteriorly to posteriorly.

**Figure 4:**
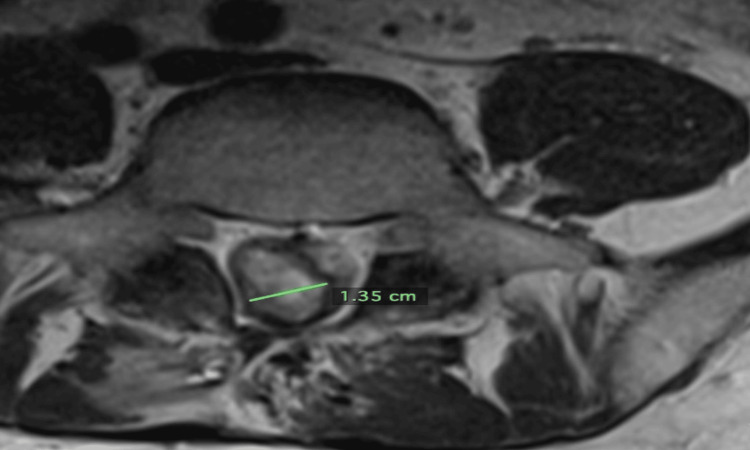
Pre-cryoablation axial image from an MRI L-spine dated May 3, 2023. The tumor measured 1.35 cm transversely.

Four months later, on May 3, 2023, an MRI L-spine with contrast was obtained, demonstrating a significant decrease in tumor volume from 10.3 cm^3^ to 2.9 cm^3^. The tumor measured 3.64 cm x 1.35 cm x 1.32 cm (Figures 3-4). The MRI demonstrated tumor enhancement consistent with residual viable tumor. The patient’s neurologicalstatus continued to decline, and his pain steadily increased. He required higher and higher doses of narcotic pain medications.

In mid-May of 2023, interventional oncology was consulted. Percutaneous image-guided cryoablation was proposed and accepted as the next therapeutic step.

Given two prior resections with early recurrence and anticipated arachnoidal adhesions, further open surgery carried elevated morbidity with uncertain incremental benefit in intradural disease. Literature on intradural extramedullary tumors notes non-trivial recurrence despite surgery and advises avoiding aggressive dissection when tightly adherent to neural structures, favoring function-preserving strategies. In our case, stereotactic radiosurgery (SRS) was constrained by spinal cord/cauda equina dose limits, making ablative dosing at the thecal sac less feasible without exceeding tolerance and risking radiation myelopathy. In contrast to heat-based ablation (RFA, microwave, laser interstitial thermal therapy), which delivers indiscriminate thermal injury in the target zone, cryoablation provides CT-visible ice-ball margins for precise coverage and tends to preserve myelin and perineurial architecture, enabling axonal regeneration and reducing the risk of permanent neurologic deficit. Contemporary series show percutaneous spine cryoablation is safe and effective for local tumor control and pain palliation, supporting its use as salvage in complex spine tumors adjacent to critical neural elements.

**Figure 5:**
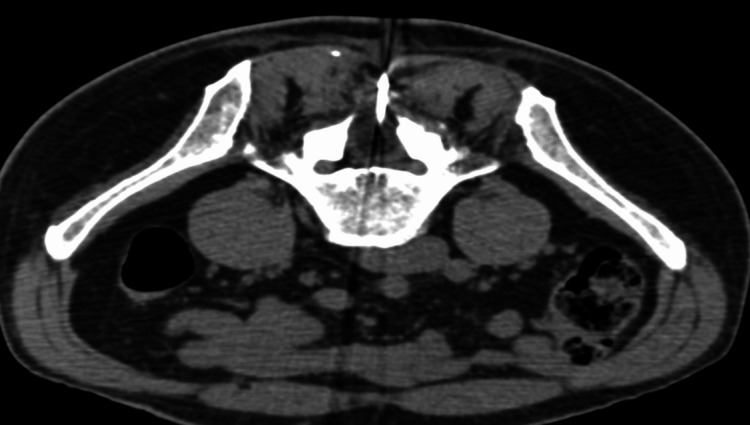
Axial image of the CT-guided cryoablation performed on June 3, 2023. The image demonstrates a cryoprobe inserted through the area of the prior lumbar laminectomy. The low-density oval-shaped area around the probe in the thecal canal represents the ice ball partially consuming the spinal tumor.

On June 3, 2023, the patient underwent percutaneous CT-guided cryoablation of the intradural extramedullary tumor (Figure 5). The anesthesia team administered an intravenous cocktail of propofol, Versed, and morphine during the four-hour case. With the patient in the prone position, an overlapping technique was used to insert three cryoprobes measuring 2.1 mm OD from a posterior approach through thesuperior, central, and inferior regions of the laminectomy site. Once all three probes were positioned to satisfaction and confirmed with imaging, three active argon gas freeze cycles and three active helium gasthaw cycles were performed. The three cryoprobes were placed and repositioned so that ultimately nine trueprobe positions were utilized from superior, middle, and inferior regions, as well as left and right lateralregions. Angling of the probe allowed for targeting of the tumor that extended into the lateral recesses. Imaging documented that all visible tumors, as well as a small peripheral rim of non-tumorous tissue wastreated and the treated tissue reached a temperature of -40 degrees Celsius and below, which is significant given that -40 degrees Celsius achieves cyto-destructive temperatures in all circumstances.

The post-op course was complicated with issues of pain control. Pre-operatively, the patient had been onopiate pain medications for 18 months. In an effort to achieve a livable level of pain, he had reached and maintained a maximum therapeutic dose that rivaled a toxic or lethal dose.

Post-operatively in the recovery room, he suffered an ebb and flow of significant pain requiring titrationof his narcotic medication. Because of his tolerance level, the dose required to control his post-operative pain was enough to initiate respiratory suppression when his pain ebbed. To further complicate matters, he suffered from poorly controlled pain-induced hypertension in the immediate post-op period. To stabilize the patient and address the issues of hypertension, intractable pain, andrespiratory suppression, the patient was placed in a CNS-suppressed state and intubated for 36 hours in the ICU. In the sedated state, his narcotics were discontinued. During the post-operative timeframe, his laboratory suggested the possibility of a cardiac event, but analysis and workup proved this to be false. His post-op course was otherwise uneventful, and he was discharged from the hospital approximately 72 hours after his surgery. His hypertension had resolved, and he was on no pain meds at discharge. Surprisingly, he did not suffer opiate withdrawal.

Several days after he was discharged, his spouse noticed long-term memory deficits. Memory loss workup included a brain MRI with contrast on August 1, 2023. The MRI was interpreted as normal. No etiology of the memory loss was found.

**Figure 6:**
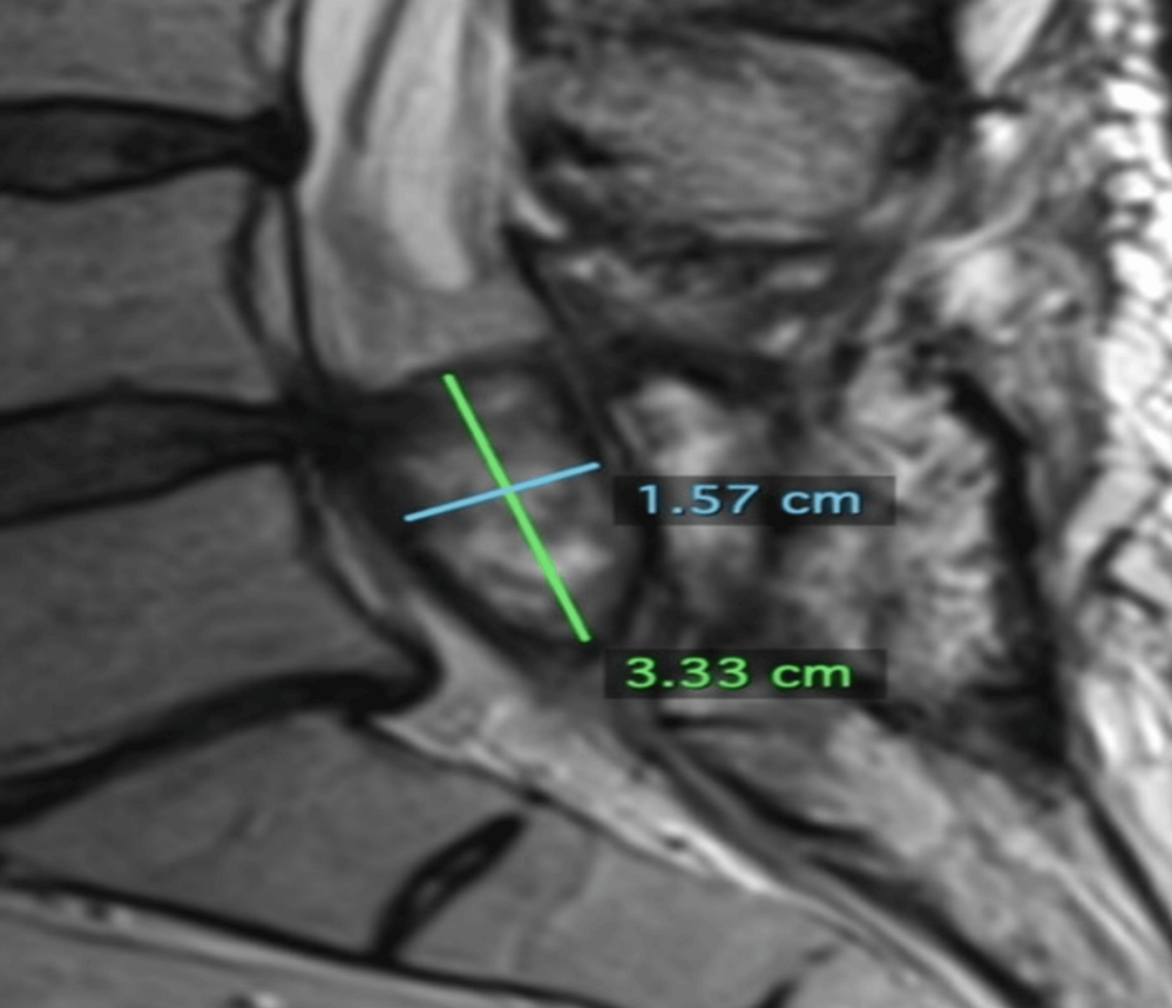
Sagittal image from an MRI L-spine with contrast obtained approximately two months post-cryoablation dated Aug 1, 2023. The tumor measures 3.33 cm superiorly to inferiorly and 1.57 cm anteriorly to posteriorly.

**Figure 7:**
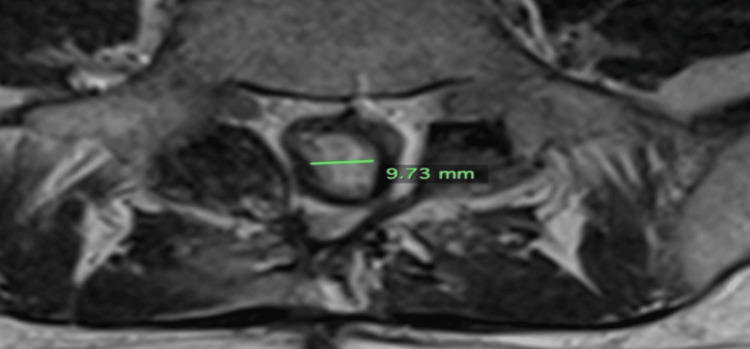
Axial image from an MRI L-spine with contrast obtained approximately two months post-cryoablation dated Aug 1, 2023. The tumor measures 0.97 cm transversely.

On August 1, 2023, two months after the cryoablation procedure, an MRI L-spine with contrast demonstrated the tumor to have decreased in size and volume, measuring 3.33 cm x 1.57 cm x 0.97 cm (2.67 cm^3^) (Figures 6-7).

**Figure 8:**
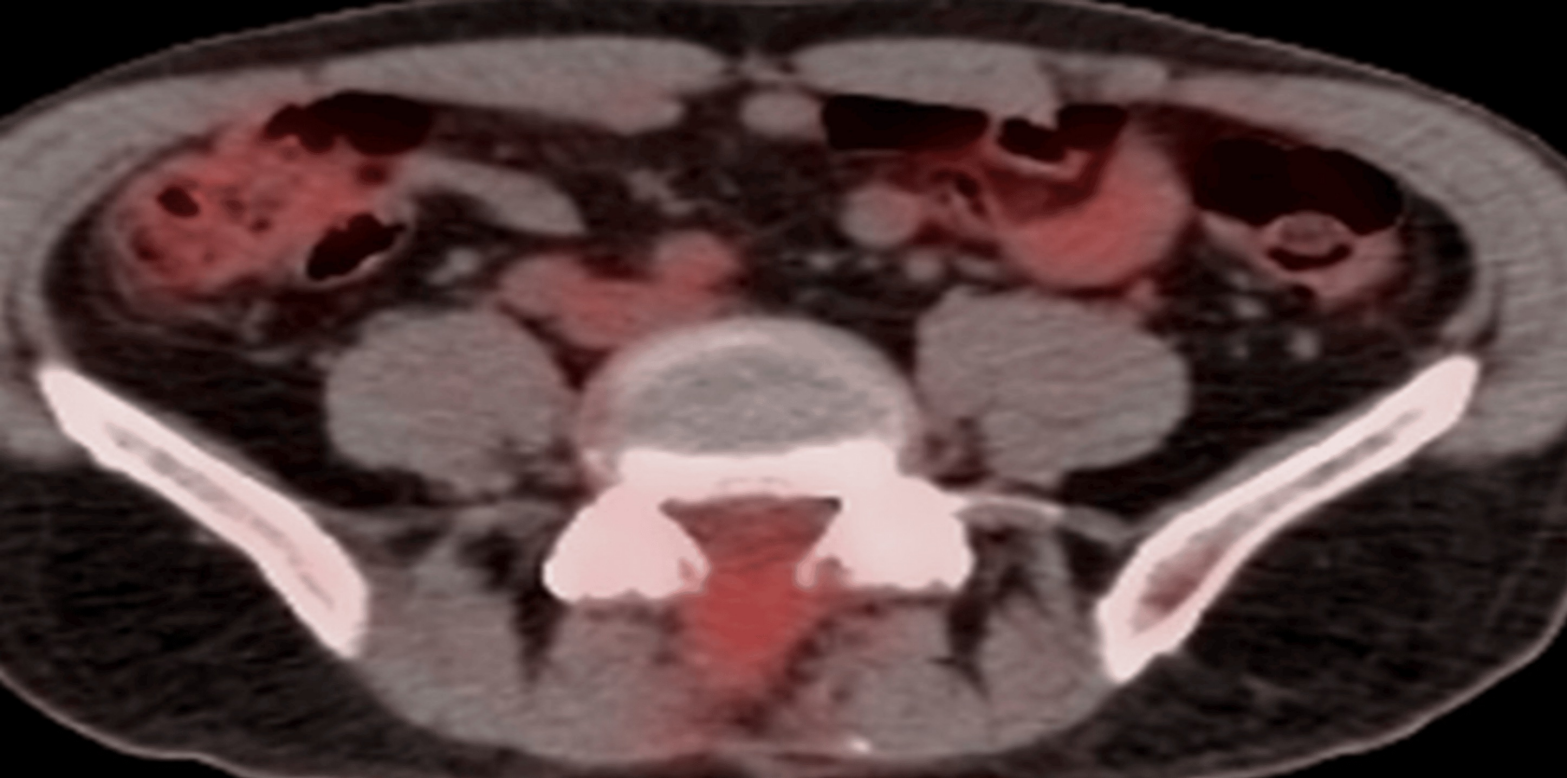
PET scan obtained September 11, 2023. Demonstrating no abnormal intrathecal fluorodeoxyglucose (FDG) uptake but increased FDG uptake in the laminectomy area is consistent with granulomatous tissue in the surgical bed.

Approximately three months after cryoablation on September 11, 2023, a whole-body FDG PETCT scan was obtained. Lumbar intrathecal FDG uptake was normal. This was consistent with non-viable cryo-ablated tumor residue. Increased FDG uptake in the extradural tissues in the region of the laminectomy was consistent with post-laminectomy granulomatous tissue (Figure 8).

As of October 2023, there had been a slight improvement in the patient’s memory; however, some deficits remained; thus, a second MRI of the brain with contrast was obtained on October 21, 2023. That study demonstrated a normal brain without evident pathology and no explanation of the memory deficit etiology. We postulate that the cause of the memory loss might be one or more small undetectable CVAs, possibly a side effect of the anesthesia or his post-operative hypertension.

**Figure 9:**
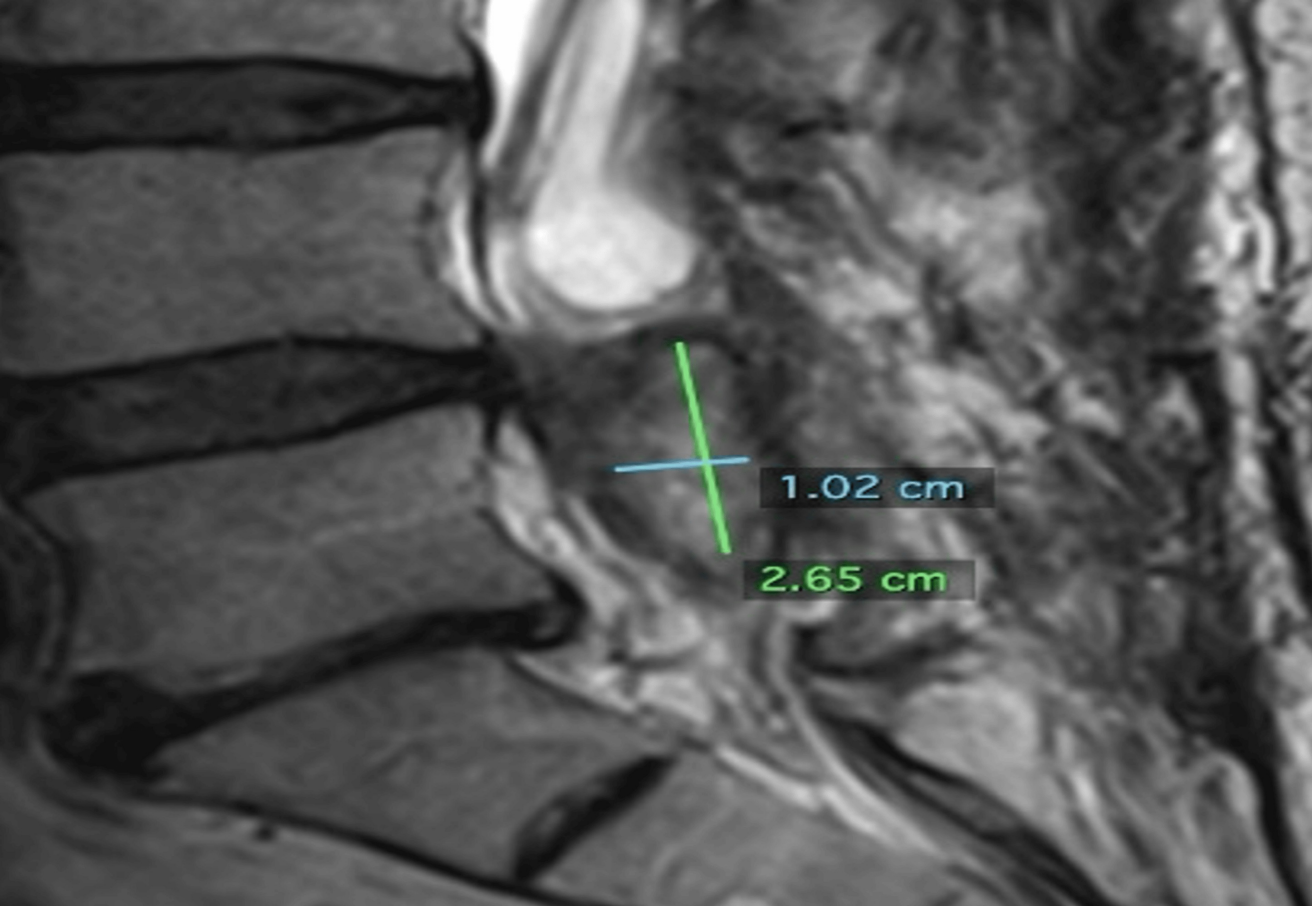
Sagittal image from an MRI L-spine with contrast dated November 7, 2023, approximately five months post cryoablation. The tumor to measure 2.65 cm superiorly to inferiorly and 1.02 cm anteriorly to posteriorly.

**Figure 10:**
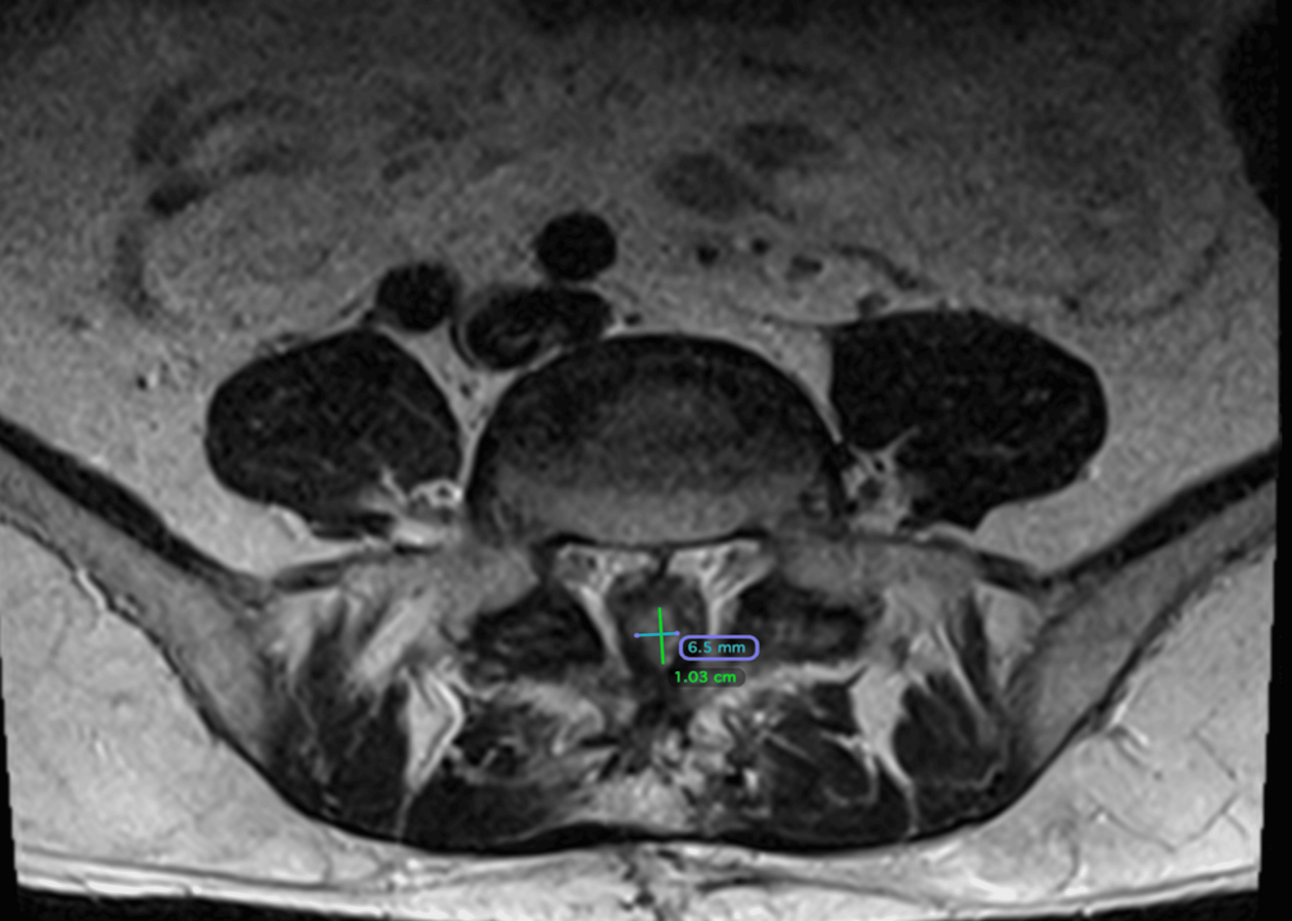
Axial image from an MRI L-spine dated June 20, 2024, approximately 13 months post cryoablation. The tumor measured 1.03 cm ant-post and 0.65 cm transversely.

Five months post cryoablation on November 7, 2023, an MRI L-spine with contrast demonstrated a significant reduction in residual tumor size and volume. The tumor measured 2.65 cm x 1.02 cm x 0.9 cm (1.27 cm^3^) (Figures 9-10).

**Figure 11:**
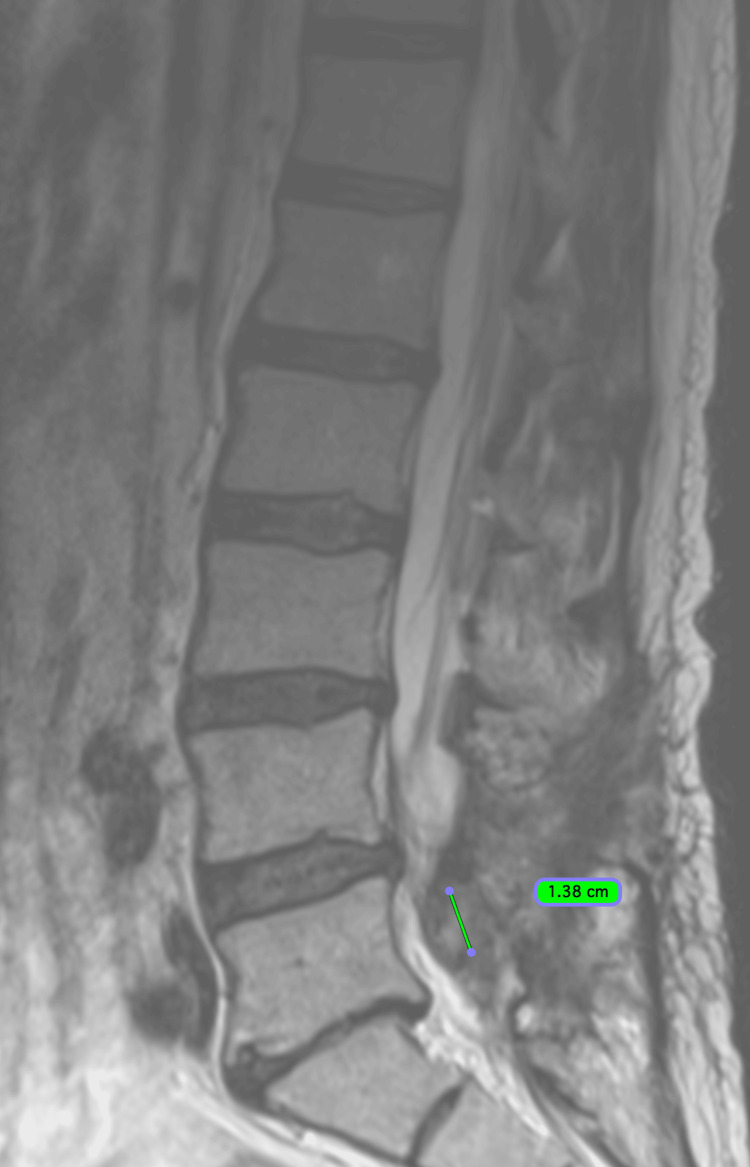
Sagittal image from an MRI L-spine with contrast dated October 10, 2024. Approximately 17 months post cryoablation, the tumor measured 1.38 cm superiorly to inferiorly.

Thirteen months post cryoablation on June 20, 2024, an MRI L-spine with contrast demonstrated the tumor to measure 1.45 cm x 1.03 cm x 0.65 cm (Figure 11).

Seventeen months post cryoablation on October 10, 2024, an MRI L-spine with contrast demonstrated the tumor to measure 1.38 cm x 0.98 cm x 0.56 cm (0.40 cm^3^).

Before undergoing cryoablation surgery in May of 2023, the patient’s overall status was severely compromised, with little quality of life. He suffered from intractable pain despite aggressive and comprehensive attempts at pain control. He suffered from severe depression and was in constant severe pain, only slightly mitigated by high doses of narcotic pain medication. He became physically dependent on the narcotic pain medications. He was incontinent of bowel and bladder and suffered from para-rectal decubitus ulcers and bilateral gluteal and lower extremity radicular pain. Severe coccydynia prevented him from sitting upright, and erectile dysfunction was complete. He was unable to walk and confined to a wheelchair.

As of November 20, 2025, 30 months after his cryoablation, the patient is back at work, having resumed his physician and management duties. His depression had long lifted. His neurological systemis significantly recovered. He is fully ambulatory, walking independently without assistance or balance aids (Figure 9). The radiculopathy and coccydynia have resolved. His decubitus ulcers had long agohealed. His colon function remains slightly improved, although he is not yet able to have a natural bowel movement on his own. His bladder dysfunction is slightly improved. His erectile dysfunction is slightly improved.

Ethical approval and patient consent

This study was conducted in accordance with institutional and international ethical standards. Ethical oversight and approval were granted by the Medical Ethics Committee. The patient provided written informed consent for participation and publication of this report and associated images. No follow-up biopsy was performed after imaging-confirmed remission, as laboratory studies and serial MRI and PET-CT evaluations demonstrated complete response at three years post-cryoablation, and the patient declined further invasive testing.

Timeline of key events

Tables 1-2 present the timeline of key events.

**Table 1:** Chronological summary of the clinical events and tumor measurements.

Date	Event Description	Tumor Dimensions	Tumor Volume
Initial Presentation and Symptoms			
2015-2016	Onset of altered bowel and bladder patterns with urgency	N/A	N/A
Sept-Oct 2021	Development of radicular buttock pain	N/A	N/A
Late Oct 2021	Cauda Equina Syndrome	N/A	N/A
Nov 24, 2021	MRI L-spine - tumor at L4	3.2 × 1.7 × 1.4 cm	3.98 cm³
Surgical Interventions			
Dec 9, 2021	First surgical resection at Stanford	N/A	N/A
Jan 10, 2022	Post-operative MRI shows recurrence	2.4 × 1.7 × 1.7 cm	3.63 cm³
Jan 14, 2022	Second surgical resection at Cedars-Sinai	N/A	N/A
Chemotherapy Treatment			
Feb 11-15, 2022	First cycle of EP chemotherapy	N/A	N/A
Feb 24, 2022	MRI shows tumor increase	4.4 × 1.9 × 1.8 cm	7.88 cm³
Mar 4, 2022	Chemotherapy discontinued	N/A	N/A
Nov 16, 2022	MRI shows tumor growth	5.7 × 2.52 × 2.05 cm	15.42 cm³
Immunoembolization Treatment			
Dec 17, 2022	Spinal artery immuno-embolization	N/A	N/A
May 3, 2023	MRI shows residual viable tumor	3.64 × 1.35 × 1.32 cm	2.9 cm³
Cryoablation Treatment and Recovery			
June 3, 2023	CT-guided cryoablation performed	N/A	N/A
Aug 1, 2023	MRI (2 months post-cryoablation)	3.33 × 1.57 × 0.97 cm	2.67 cm³
Sept 11, 2023	PET-CT: no viable tumor	N/A	N/A
Nov 7, 2023	MRI (5 months post-cryoablation)	2.65 × 1.02 × 0.9 cm	1.27 cm³
June 20, 2024	MRI (13 months post-cryoablation)	1.45 × 1.03 × 0.65 cm	0.51 cm³
Oct 10, 2024	MRI (17 months post-cryoablation)	1.38 × 0.98 × 0.56 cm	0.40 cm³
May 20, 2025	Full recovery, back at work	N/A	N/A√

**Table 2:** Tumor dimensions and response to therapy. Note tumor volumes.

Date	Study	Tumor Dimension	Tumor Volume
November 24, 2021	Pre-excision MRI L-spine	3.2 cm x 1.7 cm x 1.4 cm	3.98 cm^3^
January 10, 2022	Post-excision recurrence #1 MRI	2.4 cm x 1.7 cm x 1.7 cm	3.63 cm^3^
February 24, 2022	Post-excision recurrence #2 MRI	4.4 cm x 1.9 cm x 1.8 cm	7.88 cm^3^
November 16, 2022	Post-chemotherapy MRI/Pre-Spinal Artery Embolization	5.7 cm x 2.52 cm x 2.05 cm	15.42 cm^3^
May 3, 2023	Pre-cryoablation MRI	3.64 cm x 1.35 cm x 1.32 cm	2.9 cm^3^
August 1, 2023	Post-cryoablation 2 months MRI	3.33 cm x 1.57 cm x 0.97 cm	2.67 cm^3^
September 11, 2023	FDG-PET Scan	Not applicable	No viable tumor
November 7, 2023	Post-cryoablation 5 months MRI	2.65 cm x 1.02 cm x 0.9 cm	1.27 cm^3^
June 20, 2024	Post-cryoablation 13 months MRI	1.45 cm x 0.65 cm x 1.03 cm	0.51 cm^3^
October 10, 2024	Post-cryoablation 17 months MRI	1.38 x 0.984 x 0.564 cm	0.40 cm^3^

## Discussion

This is a case of an intradural extramedullary lumbar spine tumor causing cauda equina syndrome (CES). CES results from a variety of different pathologiescompressing and distorting the nerves of the conus medullaris at the levels of L1-L5 but classically L3-L5. CES resulting from a herniated lumbar intervertebral disc represents 45% of all CES cases [11]. Less common causes include primary and metastatic cancer, trauma with retro-pulsed bone or an epidural hematoma, infection such as diskitis or epidural abscess, and iatrogenic causes such as placement of an intra-spinous device [12]. Because the CES nerves are major sensory and motor contributors to the legs, bowel, bladder, anus, and perineum, disruptionresults in a clinical picture characterized by back pain, bilateral lower extremity radiculopathic pain, bowel and bladder muscle and sphincter dysfunction, and sexual organ dysfunction.

CNS primary tumors that occur in the intradural extramedullary spinal cord include meningioma, schwannoma, myxopapillary ependymoma, paraganglioma, solitary fibrous tumor, hemangiopericytoma, lipoma, spinal nerve sheath myxoma, sarcoma, vascular tumors, teratoma, and neurofibroma [1,3,8]. These tumors make up only 2%-4% of all central nervous system neoplasms [2].

Metastasis to the spinal column is a common occurrence; however, intradural extramedullarymetastasis is an extremely rare condition with an autopsy incidence of less than 5% [1]. Intradural extramedullary metastasis can arise from both CNS and non-CNS tissues.

CNS-sourced metastasis to the intradural extramedullary spinal cord includes glioma, glioblastoma, plastic astrocytoma, medulloblastoma, pineal body tumor, and choroid plexus tumor [1,4]. Non-CNS tumors that metastasize to the intradural extramedullary spine include lung, breast, kidney, melanoma, lymphoma, and leukemia [1]. Unlike metastasis to the vertebral column, non-CNS metastasis to the intradural extramedullary spinal cord is exceedingly rare andmake up only 0.1%-2% of all spinal cord tumors [1,3].

Diagnostic imaging plays a very important role in the evaluation of cauda equina syndrome. MRI is the most useful diagnostic imaging tool, frequently helping in the identification of the tumor; however, the imaging features are not always pathognomonic, and classification as benign or malignant based on MRI findings alone is often difficult [1].

For intradural extramedullary neoplasms threatening neurological tissues, surgery can restore and preserve neurological function. For tumors that present acutely with neurological deficits, surgery becomes the first therapeutic option. Laminectomy decompression with or without some degree of debulking to remove the compressive pathology frequently restores neurological function and significantly reduces or eliminates pain [1].

Studies comparing conservative management with surgical decompression or debulking ofsymptomatic intradural extramedullary neoplasms demonstrate decreased pain, increased quality of life, and increased survival in the surgically treated group [1].

In this case, the initial cytopathological analysis yielded a diagnosis of mature teratoma. The rapidrecurrence after the first resection prompted a second opinion of the initial pathological specimen. Thatspecimen was reclassified as a non-mature germ cell tumor with predominant immature teratoma features and transformation to sarcoma. The second surgical resection cytopathological analysis yielded the diagnosis of malignant non-seminomatous germ cell tumor with a predominance of immature teratoma. There was some agreement between the biopsies, and ultimately the consensus pathology report yielded the diagnosis as benign teratoma (immature or mature) with sarcomatous transformation.

The etiology of teratomas is unclear. In adults, a history of trauma, including iatrogenic trauma, seems to precede the clinical presentation of teratoma [6,8].

Teratomas are a multipotential cell tumor derived from at least two of the three embryonic germinal layers formed by normal organogenesis and reproductive tissues. The three germinal tissues are endoderm, mesoderm, and ectoderm [6,8].

Teratomas are classified according to their embryonic germ cell components as mature benign, immature benign, and malignant.

Mature benign teratomas are comprised of fully differentiated adult-type components, including mature cartilage, skin appendages, columnar mucosa, smooth muscle, hair and teeth, respiratory tissues, neural elements, and squamous epithelium [7-9,12].

Immature benign teratomas are tumors that typically contain components that are almost exclusively neuroepithelial and of fetal origin, except for rare cases of primitive mesenchymal tissue and renal tissue [7,8].

Malignant teratoma are tumors containing yolk sac or ectodermal sinus-derived malignant germ cell layer tissues [6,7]. These tumors commonly produce elevated levels of serum alpha-fetoprotein and have a very poor prognosis [9].

Intradural extramedullary spinal teratoma is exceedingly rare in adults, but when they occur, the conus medularis is the most common location [11,12]. A review of the literature documents a total of 146 reported cases, and only 12 of these occurred in patients over age 60 years [6]. Intradural extramedullary spinal teratomas account for only 0.2-0.5% of all spinal cord tumors [6,8,9,11]. Mature teratomas have a 2% risk of malignant transformation and a <1% risk of cystrupture [6]. Mature teratomas within the spinal cord are slow growing, with an average growthof 1.8 mm/year [6].

Surgical resection is the treatment of choice. Early diagnosis and gross total resection are correlatedwith the best surgical outcomes [8]. Because approximately 50% of teratomas are adherent to the surrounding neural structures, complete resection is often not possible. Teratoma recurrence is not uncommon; however, the rates of recurrence will depend on the histopathological characteristics of the tumor. Recurrences were found to be more common in immature and malignant teratomas [6,8,10].

Mature teratomas are relatively benign. In a recent study involving mature spinal teratomas, the recurrence rates for complete and gross resection were extremely similar (9 and 11%, respectively). This is a consequence of the slow teratoma growth and slow regrowth after incomplete resection [7,8,12]. Serial MRI exams will demonstrate the rate of growth recurrence and dictate further therapy for a particular teratoma [6,9].

The role of adjuvant therapies, including radiotherapy and chemotherapy for the remnant tumors, has not been characterized clearly. Its effects for immature and malignant teratomas also remain controversial [11]. Consideration for the use of radiation therapy and or chemotherapy in ateratoma with malignant features and cellular elements is justified [9,11].

In the past 25 years, image-guided ablation techniques and technology were developed and applied totreatment of cancer with great success [13]. The benefits of minimal invasiveness and targeted therapyusing electromagnetic and thermal energy proved to be effective in addressing some of the limitationsof prior oncologic therapies.

Thermal ablation is divided into hot and cold. The distinction has significant implications for the central nervous system. Ablation technology that utilizes temperature increase includes microwave, radiofrequency, and laser technology and results in indiscriminate tissue destruction in the targetfield once a target temperature is reached. Cells die by protein and enzyme denaturation. Ablation technology that uses a temperature decrease to crystallize water and rupture cell membranes is called cryoablation. Cryoablation has a particularly unique strength regarding the ablation of neural tissues. At or below the target temperature of -40 degrees Celsius, the nerve axons die; however, the myelin sheath cells are largely cryo-resistant and survive at the same temperature. Preservation of the myelin sheath allows for axonal regeneration at a rate of 1.5 mm/day ± 1.1 [13]. Compared to all other forms of tissue ablation, cryoablation has, at the very least, a good chance of preserving neurological function and, at best, allowing for the restoration or recovery of neural function over time, often in a matter of months [14].

This patient's neurological function has improved dramatically over the months after cryoablation. The patient now walks without any form of aid. Cryoablation of the lumbar intradural extramedullary spinal tumor resulted in the death of the tumor. The normal mechanisms responsible for dead tissue reabsorption decompressed the remaining viable neural elements in the cauda equina, resulting in a degree of neural structure salvage. In the acute and subacute timeframe, this mechanism can appear to represent the recovery of previouslylost neural function, while it likely also represents a reversal of hibernating - such as neural tissue. I also believe that this neurological recovery is from the regeneration of neural structures made possible by the above-described mechanism, that it is unique to the physiological properties of cryoablation, and that it is an axonal regeneration inside a myelin sheath. This regenerative process is not seen with other ablation technologies, such as radiofrequency, microwave, and chemical ablation. This myelin sheath-sparing property makes cryoablation superiorly suited for tumor ablation in the nervous system.

## Conclusions

This case represents a landmark achievement in the management of intradural extramedullary spinal tumors, demonstrating the first successful management of a malignant lumbar spinal teratoma using percutaneous CT-guided cryoablation. The complexity of this case underscores several critical aspects of modern neuro-oncological practice. First, it highlights the diagnostic challenges inherent in classifying these rare tumors, as evidenced by the conflicting pathological interpretations that ranged from benign mature teratoma to malignant non-seminomatous germ cell tumor with sarcomatous transformation. Second, it illustrates the limitations of conventional therapies, as both surgical resection and systemic treatments failed to control the disease. Most significantly, this case demonstrates the unique advantages of cryoablation in the treatment of spinal tumors, particularly its ability to preserve myelin sheaths while destroying tumor cells, thereby allowing for the remarkable neurological recovery observed in this patient. The patient's transformation from wheelchair-bound disability with intractable pain to full ambulation and return to professional practice represents not just a therapeutic success but a validation of innovative approaches to complex spinal pathology. This case should encourage further investigation into the role of cryoablation for similar challenging cases and may represent a paradigm shift in how we approach intradural extramedullary tumors that are refractory to conventional treatments.
